# Impact of creatine supplementation on kidney health: a systematic review and meta-analysis

**DOI:** 10.1007/s11255-026-05287-x

**Published:** 2026-07-27

**Authors:** Artur de Souza Almeida, Leonardo Olimpio Dias da Silva, Bianca Namie Abe Takahasi, Bruno Damico Terada, Felipe Giraldo Alvarez Gonçalves, Breno Cordeiro Porto, Rodrigo Afonso da Silva Sardenberg, Carlo Camargo Passerotti, Ronaldo Soares Maia, Farid Samaan, Maxton Thoman, Wilson R Molina, Jose Arnaldo Shiomi da Cruz

**Affiliations:** 1 Department of Urology, Federal University of São Paulo (UNIFESP), Sao Paulo, Brazil; 2 Research Institute, Hapvida NotreDame, Sao Paulo, Brazil; 3 Department of Urology, The University of Kansas Health System, Kansas City, KS USA; 4 Department of Urology, Ninth of July University, Sao Bernardo do Campo, Brazil

**Keywords:** Creatine supplementation, Kidney health, Hemodialysis, Serum creatinine, Glomerular filtration rate

## Abstract

**Purpose:**

To evaluate the safety of creatine supplementation on kidney health in healthy individuals and patients with CKD.

**Methods:**

The MEDLINE, Embase, Cochrane Library, and Google Scholar databases were systematically searched for RCTs from inception to March 2025. Studies comparing creatine supplementation with placebo in human subjects that assessed kidney health parameters were selected by 2 independent reviewers following PRISMA guidelines. A random-effects model was used to pool mean differences with 95% CI. The primary outcome was serum creatinine level. Secondary outcomes included GFR assessed by creatinine-based methods and by Cr-EDTA, serum urea, albuminuria, proteinuria, and urinary creatinine.

**Results:**

Twenty-six studies (1036 participants) were included. Creatine supplementation was associated with a significant increase in serum creatinine levels (MD, 0.14 mg/dL; 95% CI 0.05–0.22; *P* = .002; *I*^2^ = 93.9%) and a reduction in GFR assessed by creatinine-based methods (MD, –10.75 mL/min; 95% CI –17.48 to –4.02; *P* = .002; *I*^2^ = 0%). No significant differences were observed when assessed by Cr-EDTA (MD, 5.89 mL/min; 95% CI –0.30 to 12.08; *P* = .06; *I*^2^ = 6.3%) or in serum urea, albuminuria, proteinuria, and urinary creatinine. In the hemodialysis subgroup, creatine supplementation was associated with a significant increase in serum creatinine; conversely, serum urea levels were significantly reduced.

**Conclusions:**

Creatine supplementation is associated with increased serum creatinine levels and reduced glomerular filtration rate estimated by creatinine-based methods in healthy individuals and patients with chronic kidney disease. These findings likely reflect altered creatinine metabolism rather than kidney injury, given the absence of changes when assessed using Cr-EDTA.

**Trail registry:**

CRD420251015042.

**Supplementary Information:**

The online version contains supplementary material available at https://doi.org/10.1007/s11255-026-05287-x.

## Introduction

Creatine is a nitrogenous organic compound synthesized endogenously from amino acids, though it can also be obtained exogenously through dietary sources, primarily animal products [1]. In 1992, Harris et al. [2] published a seminal study demonstrating the effectiveness of creatine supplementation in elevating its intramuscular levels. Since then, numerous studies have investigated its efficacy as an ergogenic aid, particularly for improving performance during high-intensity, short-duration exercise and increasing muscular strength [3].

In recent years, evidence has expanded beyond physical performance, with growing interest in its potential clinical applications [4–6]. Despite its widespread use and general perception of safety in healthy populations, concerns persist regarding the long-term effects of creatine on kidney health. Consequently, creatine use has traditionally been discouraged in individuals with impaired kidney health, largely due to concerns and elevations in serum creatinine concentrations [7].

While position stands from organizations such as the International Society of Sports Nutrition [8] have consistently described creatine supplementation as safe in healthy populations, these conclusions are largely derived from studies not primarily designed to assess kidney health outcomes. Conversely, kidney-focused clinical guidelines, including KDIGO [9], do not provide specific recommendations regarding creatine use, reflecting the absence of consolidated evidence rather than established safety or harm. Given that creatine supplementation directly influences creatinine metabolism [10], mere reliance on serum creatinine as a surrogate marker introduces substantial uncertainty in the interpretation of kidney safety profile [11]. Prior reviews have been limited by heterogeneous study designs, reliance on serum creatinine as a sole renal surrogate, and the absence of stratification by measurement methodology, and the absence of stratification by measurement methodology or by clinical population. Therefore, the present systematic review and meta-analysis aim to provide a more rigorous evaluation of the effects of creatine supplementation on kidney health through multiple outcomes in both healthy individuals and patients receiving hemodialysis.

## Methods

### Search strategy

This systematic review and meta-analysis followed the Cochrane Handbook for Systematic Reviews of Interventions [12] and the PRISMA guidelines [13]. We searched MEDLINE (via PubMed), Embase, the Cochrane Library, and Google Scholar for studies published from inception to March 2025. Web of Science was not searched independently, given its substantial overlap with MEDLINE and Embase. Google Scholar served as a complementary source for grey literature identification. Eligible studies were Randomized Clinical Trials (RCTs) evaluating the effects of creatine supplementation on kidney-related outcomes compared with placebo. The full search strategy is provided in eMethods in Supplementary 1. The study protocol was registered in PROSPERO (CRD420251015042).

### Eligibility criteria for study selection

The authors included studies that met the following criteria: (1) involved human participants; (2) were designed as RCTs; and (3) implemented a creatine supplementation protocol accompanied by pre- and post-intervention assessment of at least one outcome measure related to kidney function.

We excluded studies for the following reasons: (1) non-human studies; (2) absence of reported outcomes of interest; (3) non-RCT design; and (4) use of confounding co-interventions. Studies combining creatine with additional supplements were excluded if: (a) the co-intervention was not equally distributed across both groups, precluding isolation of creatine's effects; or (b) the co-intervention carried known nephrotoxic potential (e.g., anabolic steroids), regardless of allocation. No restrictions were applied regarding participant age, comorbidity status, or publication language of the study.

### Endpoints

The primary endpoint of this study was serum creatinine, selected as it is the most widely used biomarker of GFR in both research and clinical settings.

Secondary endpoints included urea levels, albuminuria, proteinuria, urinary creatinine levels, and GFR, measured either by Cr-EDTA or creatinine-based methods (e.g., Cockcroft-Gault formula or 24-h creatinine clearance).

### Screening

After removing duplicates with EndNote online™ 20 [14], two reviewers independently screened titles and abstracts. Disagreements were resolved by a third reviewer. Subsequently, a full-text review was conducted. No automation tools were used.

### Data extraction and quality assessment

Two reviewers independently extracted data, assessed risk of bias and evaluated certainty of evidence using a predefined protocol, with disagreements resolved by a third reviewer. This process applied equally to data extraction, risk of bias assessment, and GRADE evaluations. Risk of bias was assessed using ts = he Cochrane RoB 2 tool [15]. Following Cochrane recommendations [12], certainty of evidence of the selected seven most relevant outcomes was conducted using the GRADE approach [16].

### Subgroups selection

Serum creatinine and serum urea data were stratified into four subgroups: (1) short-term supplementation (≤ 7 days); (2) loading plus maintenance protocols (high-dose loading phase followed by a lower maintenance dose); (3) long-term supplementation without a loading phase (> 7 days of consistent daily dosage); and (4) supplementation in hemodialysis patients.

GFR outcomes were stratified by assessment methods: (1) studies utilizing creatinine-based estimates (e.g., Cockcroft-Gault formula, 24-h clearance), and (2) studies employing measured GFR via Cr-EDTA.

### Statistical analysis

For studies including multiple intervention–placebo pairs, each pair was analyzed independently. In crossover trials, within-subject comparisons were used. For trials with multiple intervention arms sharing a single placebo group, the placebo sample was proportionally divided to avoid double-counting.

Continuous outcomes were analyzed as mean differences (MD) with 95% confidence intervals (CI), using the standard deviation of the change, derived from pre- and post-intervention standard deviations. A correlation coefficient (*r*) of 0.5 was imputed for studies that did not report this value, as recommended in the Cochrane Handbook (version 6.3) [12]. Pooled estimates were calculated with the random-effects model. Results were considered to exhibit considerable heterogeneity at *I*^2^≥ 30%. In these cases, robustness and heterogeneity assessment analyses (leave-one-out [LOO] sensitivity analyses, Baujat plots, meta-regressions) were performed. If substantial heterogeneity persisted, univariate and multivariate meta-regression analyses were performed, including potential study-level covariates such as body mass index (BMI), mean age, follow-up duration, creatine dosage, and physical activity status (active *vs.* inactive individuals), to investigate whether these factors could explain the residual heterogeneity.

Statistical analyses were conducted using RStudio [17], and a Trial Sequential Analysis (TSA) was performed using the TSA software [18]. Publication bias was assessed using contour-enhanced funnel plots and the Egger regression test, in accordance with Cochrane Handbook [12] recommendations, and only for outcomes with ten or more contributing studies—the minimum threshold below which these tests lack adequate statistical power to reliably detect small-study effects.

## Results

### Study selection and characteristics

**Fig. 1:**
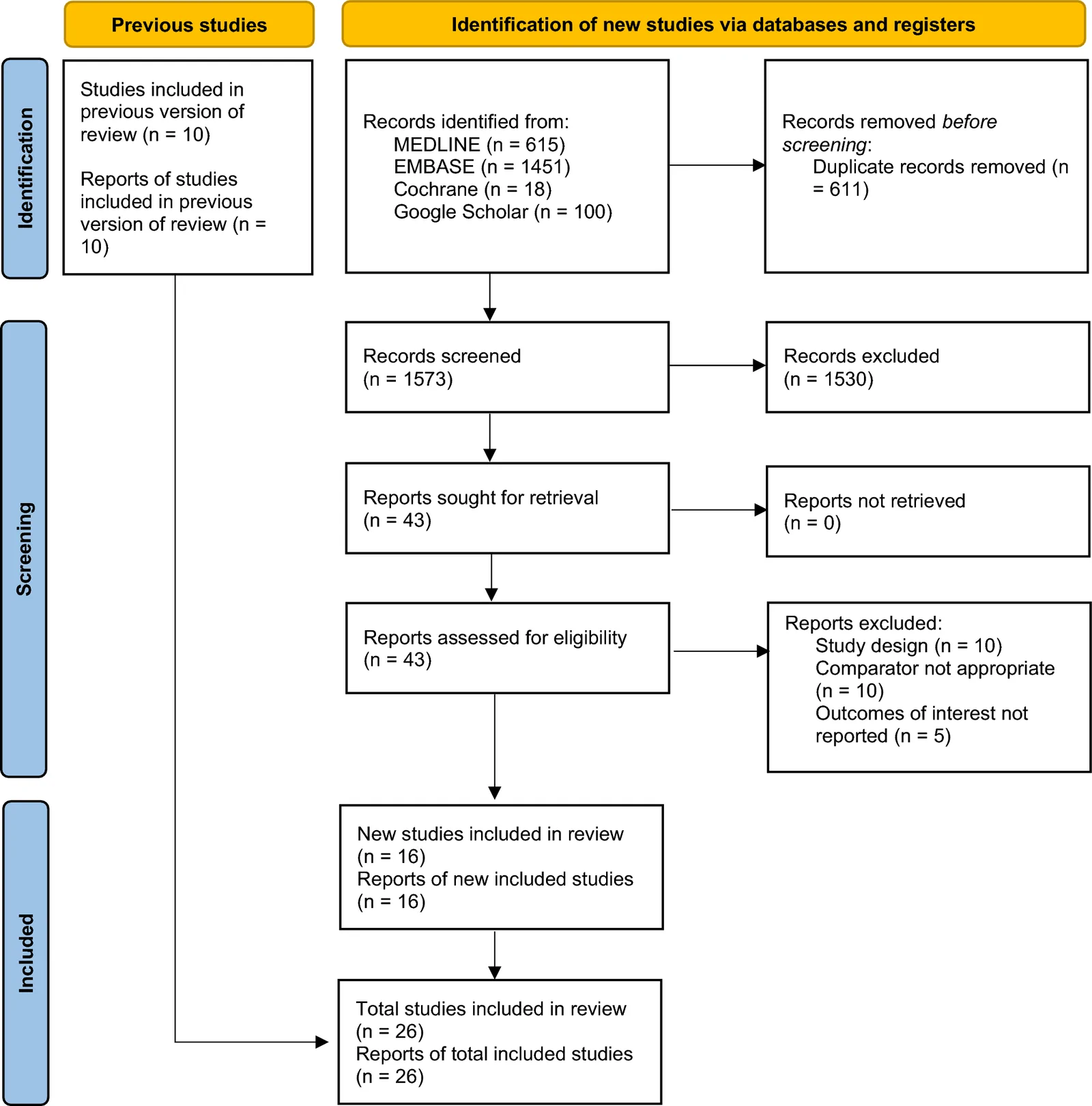
PRISMA flow diagram of the study selection process

After the systematic search, 2362 articles were retrieved. Once duplicates were removed and screening was completed, 26 RCTs [19–44] were deemed relevant and included in the analysis (Fig. 1), totalizing 1036 participants. Of these, 565 participants were evaluated pre- and post-creatine supplementation, and 471 participants were evaluated pre- and post-placebo administration. For an overview of the patient demographics across all included studies, please refer to the Table 1. A summary of creatine supplementation protocols is provided in eTable 2 in Supplement 1.

**Table 1:** Baseline characteristics

Study	Patients number		Mean age + SD		BMI	
	Creatine	Placebo	Creatine	Placebo	Creatine	Placebo
Almeida, 2013 [20]	9	9	22.7 ± 3.0	24.2 ± 3.7	25.8 ± 3.6	25.2 ± 4.6
Alves, 2013 [19]	15	13	48.7 ± 8.4	49.0 ± 10.1	28.1 ± 4.8	26.7 ± 5.1
Armentano, 2007 [22]	18	17	27.38 ± 4.74	25.94 ± 2.53	24.2 ± 3.8	25.0 ± 5.7
Bender, 2008 [21]	31	17	60.0 ± 9.4	58.7 ± 11.3	NS	NS
Bennet, 2001 [23]	8	8	23.1 ± 1.5	28.4 ± 3.3	25.3 ± 0.7	25.7 ± 1.1
Carvalho 0.03 g/kg, 2011 [24]	12	6	24.3 ± 4.9	23.0 ± 3.2	23.7 ± 2.6	22.4 ± 2.3
Carvalho 5 g, 2011 [24]	11	6	25.2 ± 7.4	23.0 ± 3.2	22.0 ± 2.2	22.4 ± 2.3
Dalton, 2017 2 g CNL	28	14	21.6 ± 3.7	21.6 ± 3.7	24.8 ± 4.4	24.8 ± 4.4
Dalton, 2017 4 g CNH	28	14	21.6 ± 3.7	21.6 ± 3.7	24.8 ± 4.4	24.8 ± 4.4
de Oliveira Vilar Neto, 2020 3 g [35]	10	5	24.4 ± 6.1	21.6 ± 3.0	25.7 ± 3.0	24.2 ± 1.5
de Oliveira Vilar Neto, 2020 5 g [35]	10	5	21.4 ± 2.8	21.6 ± 3.0	24.0 ± 2.4	24.2 ± 1.5
Domingues, 2020 [36]	14	15	64 ± 10	64 ± 8	26.7 ± 6.5	28.7 ± 3.1
Galvan, 2016 CM	13	4	21 ± 3	21 ± 3	24.8 ± 6.9	24.8 ± 6.9
Galvan, 2016 CNH	13	3	21 ± 3	21 ± 3	24.8 ± 6.9	24.8 ± 6.9
Galvan, 2016 CNL	11	4	21 ± 3	21 ± 3	24.8 ± 6.9	24.8 ± 6.9
Gotshalk, 2002 [37]	10	8	65.4 ± 1.5	65.7 ± 2.0	27.1 ± 1.0	27.7 ± 1.2
Groeneveld, 2005 [38]	81	81	57.1 ± 11.2	58.4 ± 10.9	NS	NS
Gualano, 2008 [40]	8	9	24.2 ± 5.0	24.6 ± 4.2	23.1 ± 4.5	24.0 ± 3.2
Gualano, 2011 [39]	9	12	57.5 ± 5.0	56.4 ± 8.2	31.6 ± 0.9	33.6 ± 2.0
Hayashi, 2014 [41]	15	15	15 ± 2	15 ± 2	21.6 ± 3.8	21.6 ± 3.8
Kreider, 1998 [42]	11	14	19.9 ± 0.3	19.9 ± 0.3	29 ± 1.4	29 ± 1.4
Lobo, 2015 [43]	56	53	58 ± 5	58 ± 6	27.6 ± 4	27.5 ± 3.6
Lugaresi, 2013 [26]	12	14	24 ± 3	27 ± 5	24.8 ± 1.6	24.7 ± 2.9
Lyoo, 2012 [27]	25	27	45.7 ± 12.7	47.5 ± 9.5	24.2 ± 2.7	23.1 ± 3.1
Marini, 2020 [29]	19	14	41.86 ± 3.32	41.79 ± 2.72	22.8 ± 1.4	21.9 ± 1.3
Marini, 2024 [28]	14	21	52.9 ± 15.6	52.8 ± 17.2	25.5 ± 4.3	25.0 ± 2.8
Mihic, 2000 [31]	15	15	23.1 ± 2.4	21.7 ± 1.7	24.8 ± 4.5	23.9 ± 4.8
Neves, 2011 [30]	13	11	59 ± 3	57 ± 3	28.5 ± 3.8	29.7 ± 3.2
Robinson, 2000 – CrLoad [33]	7	7	23 ± 4	24 ± 5	22.4 ± 2.1	21.3 ± 0.9
Robinson, 2000—CrLoad + G [33]	6	6	24 ± 3	22 ± 2	25.6 ± 6.3	21.7 ± 2.0
Robinson, 2000 – CrMaint [33]	7	3	26 ± 8	NS	23.3 ± 1.9	24.5 ± 3.6
Robinson, 2000—CrMaint + Ex [33]	9	3	27 ± 6	28 ± 5	23.4 ± 3.2	24.5 ± 3.6
Solis, 2016 [34]	7	8	13 ± 4	13 ± 4	21.9 ± 8.1	21.5 ± 7.5
Volek, 2001 [44]	10	10	23.0 ± 1.0	23.1 ± 1.0	25.1 ± 1.1	25.2 ± 1.8

*BMI* body-mass index, *CM* creatine monohydrated, *CNH* creatine nitrate high dose, *CNL* creatine nitrate low dose, *CrLoad* creatine load, *CrLoad* + *Gl* creatine load + glucose, *CrMaint* creatine maintenance, *CrMaint* + *Ex* creatine maintenance + exercise

### Meta-analysis

#### Primary endpoint

**Fig. 2:**
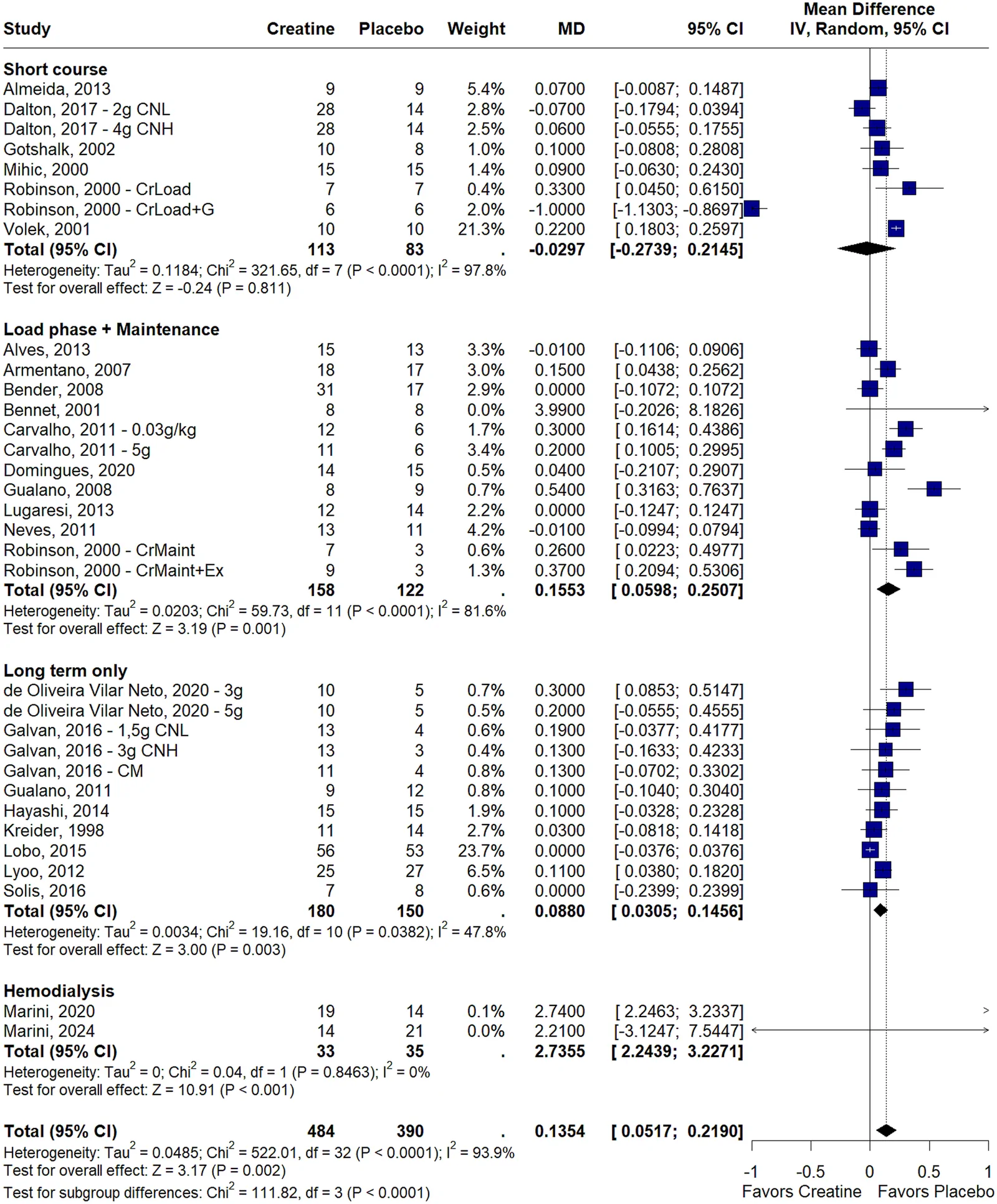
Forest plot of the effect of creatine supplementation on serum creatinine levels

When comparing the change in serum creatinine levels between patients receiving creatine and those receiving placebo, a statistically significant increase was observed in the intervention group, indicating a greater change in serum creatinine compared to the placebo group (MD 0.14 mg/dl; 95% CI 0.05–0.22; *P* = 0.003; *I*^2^ = 93.3%; *Q*-test *P* < 0.0001) (Fig. 2). Subgroup analyses confirmed this effect in the load phase plus maintenance regimen (MD 0.16 mg/dl; 95% CI 0.06–0.25; *P* = 0.001; *I*^2^ = 81.6%; *Q*-test *P* < 0.0001), in long-term use (MD 0.09 mg/dl; 95% CI 0.03–0.15; *P* = 0.003; *I*^2^ = 47.8%; *Q*-test *P* = 0.0382; certainty: High), and in the hemodialysis subgroup (MD 2.74 mg/dl; 95% CI 2.24–3.23; *p* < 0.0001; *I*^2^ = 0%; *Q*-test *P* = 0.8463; certainty: High), all favoring placebo. In contrast, the short-course subgroup did not demonstrate a significant difference between groups (MD −0.07; 95% CI −0.37 to 0.23; *P* = 0.668 *Q*-test *P* < 0.0001; *I*^2^ = 97.4%).

Given the substantial heterogeneity observed, sensitivity analyses and meta-regressions were performed (eFigures 2—12 in Supplement 1). In the load plus maintenance subgroup, BMI emerged as a significant moderator in multivariate analysis, explaining most of the heterogeneity. In the long-term subgroup, heterogeneity was primarily driven by a single outlier study (Lobo et al., 2015), whose exclusion reduced *I*^2^ to 0%.

A trial sequential analysis (eFigure 13 in Supplement 1) performed after exclusion of the outlier study showed that the Z-curve crossed the O’Brien–Fleming boundary, with the accumulated sample size exceeding the required information size.

#### Secondary outcomes

**Fig. 3:**
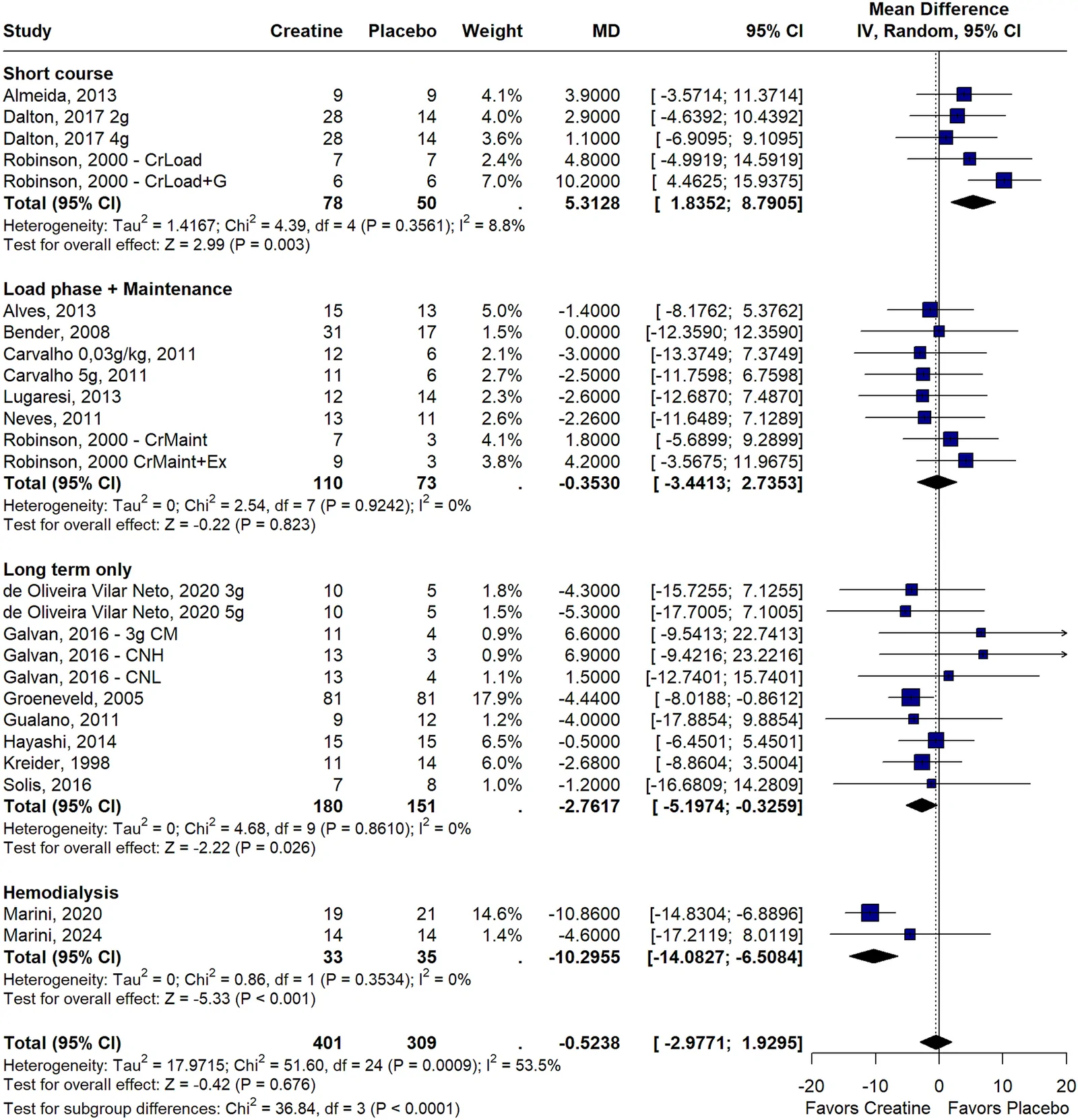
Forest plot of the effect of creatine supplementation on serum urea levels

Regarding the secondary outcomes, no statistically significant differences were identified in the overall analysis for serum urea (MD −0.52 mg/dl; 95% CI −2.97–1.93; *P* = 0.676; *I*^2^ = 53.5%; *Q*-test *P* = 0.0009; certainty: High) or in the load plus maintenance subgroup (MD −0.35 mg/dl; 95% CI −3.44 to 2.74; *P* = 0.823; *Q*-test *P* = 0.9242; *I*^2^ = 0%). In the short-term subgroup, creatine supplementation was associated with higher serum urea levels compared with placebo (MD 5.31 mg/dL; 95% CI 1.84–8.79; *P* = 0.003; *I*^2^ = 8.8%; *Q*-test *P* = 0.3561). In contrast, lower serum urea levels were observed in the long-term (MD − 2.76 mg/dL; 95% CI  − 5.19 to − 0.33; *P* = 0.026; *I*^2^ = 0%; *Q*-test *P* = 0.8610) and hemodialysis subgroups (MD − 10.30 mg/dL; 95% CI  − 14.08 to − 6.51; *P* < 0.001; *I*^2^ = 0%, *Q*-test *P* = 0.3534) (Fig. 3).

**Fig. 4:**
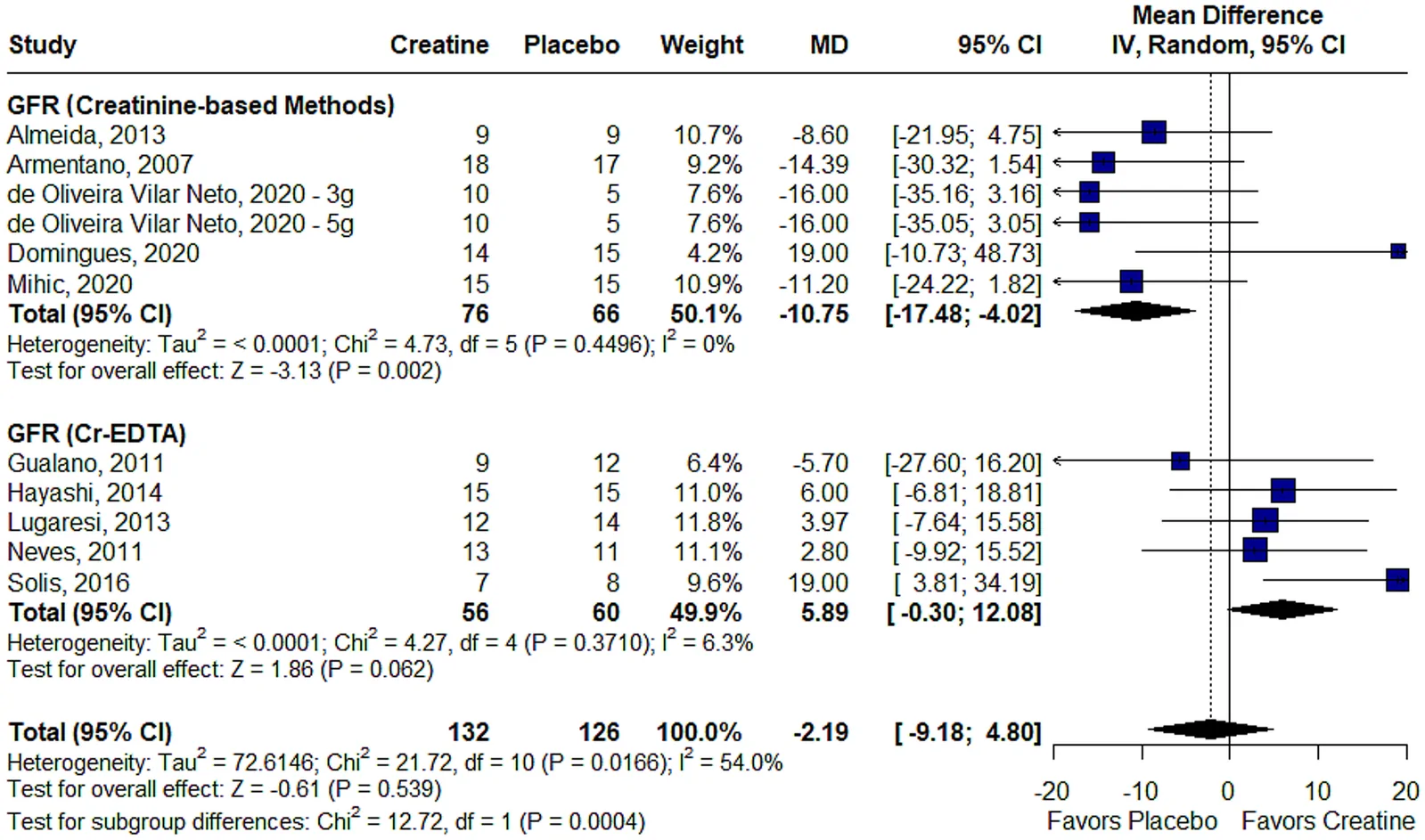
Forest plot of the effect of creatine supplementation on glomerular filtration rate

Regarding GFR, a statistically significant reduction was observed exclusively for GFR estimated using creatinine-based methods (MD − 10.75 mL/min; 95% CI − 17.48 to − 4.02; *P* = 0.002; *I*^2^ = 0%; *Q*-test *P* = 0.4496). No significant differences were detected for overall GFR (MD − 2.19 mL/min; 95% CI − 9.18 to 4.80; *P* = 0.539; *I*^2^ = 54.0% *Q*-test *P* = 0.0166; certainty: Moderate) or for GFR measured by Cr-EDTA clearance (MD 5.89 mL/min; 95% CI − 0.30 to 12.08; *P* = 0.062; *I*^2^ = 6.3%; *Q*-test P 0.3710) (Fig. 4).

No statistically significant differences were observed for other secondary outcomes, including urinary creatinine (MD − 0.09 g/24 h; 95% CI − 0.17 to 0.35; *P* = 0.484; *I*^2^ = 67.5%; *Q*-test *P* = 0.0089; certainty: Moderate), proteinuria (MD 0.01 g/24 h; 95% CI − 0.03 to 0.05; *P* = 0.633; *I*^2^ = 0%; *Q*-test *P* = 0.4716; certainty: Moderate), and albuminuria (MD 0.41 mg/L; 95% CI − 2.09 to 2.90; *P* = 0.748; *I*^2^ = 77.5%; *Q*-test *P* = 0.0013; certainty: Very Low) (eFigures 14—16 in Supplement 1).

Sensitivity analyses were conducted for urinary creatinine and albuminuria (eFigures 17—20 in Supplement 1). For urinary creatinine, heterogeneity was resolved after exclusion of a single influential study [44]. In contrast, heterogeneity in albuminuria persisted despite sensitivity and meta-regression analyses.

### Risk of bias and GRADE assessment

Of the 26 included studies, eleven were judged to present an overall low risk of bias. Fourteen studies raised some concerns, mostly related to the selection of the reported result (domain D5). Finally, one study—*Robinson *et al*.* [33]—was judged as having a high overall risk of bias, especially for concerns that arose from issues in the randomization process (eFigure 21 in Supplement 1). The certainty of evidence assessment and summary of findings are presented in eTable 3 in Supplement 1.

Publication bias was assessed for outcomes with ten or more contributing studies, per Cochrane Handbook recommendations. Funnel plot analyses for serum creatinine (eFigure 23 in Supplement 1), GFR (eFigure 24 in Supplement 1), and serum urea (eFigure 22 in Supplement 1) revealed no significant asymmetry. Egger tests confirmed the absence of meaningful small-study effects for serum creatinine (*t* = 0.85, df = 31, *P* = 0.401; bias estimate = 1.02, SE = 1.19), GFR (*t* =  − 0.32, df = 9, *P* = 0.757; bias estimate =  − 0.62, SE = 1.96), and serum urea (*t* = 1.87, df = 23, *P* = 0.074; bias estimate = 1.27, SE = 0.68), suggesting a low risk of publication bias across these outcomes.

## Discussion

Creatine is one of the most extensively studied dietary supplements [45], with well-established ergogenic efficacy and expanding clinical applications [4–6].Despite its widespread use, concerns regarding renal safety persist, fueled largely by anecdotal reports and case studies arising in the context of supratherapeutic doses, nephrotoxic co-interventions, or pre-existing kidney impairment—settings in which an independent causal role for creatine could not be established [10, 11, 46, 47].

In fact, creatine supplementation was associated with a statistically significant increase in serum creatinine in the pooled analysis, particularly in longer-duration studies. Trial sequential analysis further supports the reliability of this result, suggesting that the current body of evidence is unlikely to represent a spurious finding due to random error. This finding is consistent with the current consensus in the literature that, since creatinine is the end product of creatine metabolism, chronic creatine supplementation naturally leads to increased serum creatinine levels [48, 49]. However, this does not indicate kidney damage; rather, it reflects the augmented non-enzymatic cyclization of the expanded creatine pool to creatinine—a metabolic phenomenon independent of renal filtration or tubular function [11, 47, 50].

Serum creatinine levels are influenced not only by kidney excretion but also by muscle mass and creatine availability. In the loading plus maintenance subgroup, meta-regression identified BMI as a significant moderator of serum creatinine variation, with higher BMI associated with smaller increases following supplementation, accounting for a substantial proportion of the observed heterogeneity. This inverse association is likely multifactorial, reflecting a larger volume of distribution in individuals with greater body mass [51, 52] and, in some cases, higher glomerular filtration rates [53–56]. Taken together, these findings reinforce that serum creatinine may be an unreliable marker of kidney health in the context of creatine supplementation [47, 57], underscoring the importance of complementary biomarkers for a more physiologically accurate assessment.

Similar to serum creatinine, serum urea levels are influenced by non-kidney factors, including protein intake and hydration status [55]. In the overall analysis, creatine supplementation was not associated with significant changes in serum urea levels. However, subgroup analyses revealed a transient increase in urea during short-term interventions, whereas long-term and hemodialysis subgroups showed modest reductions. These findings should be interpreted cautiously, as they more likely reflect adaptive changes in nitrogen metabolism rather than alterations in kidney health. A biochemically plausible explanation involves feedback inhibition of endogenous creatine synthesis, which may alter arginine utilization and downstream nitrogen metabolism, contributing to early changes in urea handling [58]. Conversely, longer-term reductions in serum urea may be consistent with enhanced nitrogen retention associated with creatine supplementation [2, 56], particularly when combined with resistance training.

Regarding secondary outcomes, a statistically significant reduction in GFR was observed exclusively in studies employing creatinine-based estimation methods. This finding should be interpreted with caution, as creatine supplementation increases serum creatinine levels [40], leading to a spurious reduction in estimated GFR that does not reflect true kidney impairment. In contrast, no significant changes were observed in studies assessing GFR using Cr-EDTA clearance, an exogenous marker that is freely filtered and minimally reabsorbed, and which is considered a reference method for GFR measurement in this context [39, 59, 60]. Additionally, no significant differences were detected in other markers of kidney damage. Taken together, the stability of measured GFR and the absence of kidney injury markers support the kidney safety of creatine supplementation.

In patients with chronic kidney disease (CKD), creatine deficiency resulting from impaired kidney synthesis and dialysis-related losses is associated with sarcopenia, fatigue, reduced functional capacity, and muscle cramps [61, 62]. Consequently, creatine supplementation has been investigated to restore intracellular creatine stores and improve muscle performance [63]. However, analogous to low-protein dietary restrictions used to mitigate hyperfiltration in CKD [11], creatine, a nitrogen-containing compound, could theoretically increase kidney nitrogen load and promote hyperfiltration. Experimental studies in animal models of induced or genetic kidney disease demonstrate that creatine supplementation does not accelerate kidney failure [64]. To date, only two RCTs [28, 29] have directly evaluated creatine supplementation in hemodialysis patients, reporting no major adverse events or electrolyte disturbances. However, interpretation of findings in this subgroup warrants caution. Residual renal function—a clinically meaningful but difficult-to-quantify parameter in dialysis patients—may be confounded by creatine-induced elevations in serum creatinine. Additionally, protein-energy malnutrition is prevalent in this population, and the theoretical nitrogenous load from creatine supplementation, although not reflected in the available urea data, has not been systematically evaluated. Both studies were further limited by small sample sizes, precluding definitive risk exclusion. Our findings are consistent with previous reviews and meta-analyses supporting the kidney safety of creatine supplementation, including those by Longobardi (2023) [11], Correia (2023) [65], Silva (2019) [66], and Naeini (2025) [67]. However, unlike the narrative reviews by Longobardi and Correia, the present study provides a quantitative synthesis through meta-analyses, allowing stronger inference and clinically meaningful stratification. In addition, the systematic and predefined search strategy minimized the risk of selection bias in study identification. Compared with Naeini (2025)—the most comprehensive prior systematic review and meta-analysis, which included 12 prospective studies (10 RCTs)—our screening encompassed 26 studies, all randomized controlled trials. We further expanded subgroup analyses by follow-up duration and applied more rigorous statistical approaches to reduce heterogeneity and strengthen sensitivity analyses, enabling a more precise evaluation of kidney effects across different creatine supplementation protocols. In addition, methods for GFR assessment varied across prior studies: Silva (2019) relied on creatinine clearance as a surrogate for eGFR, and Naeini (2025) did not distinguish creatinine-based from non–creatinine-based measures, potentially leading to false-positive interpretations of kidney dysfunction. Finally, we incorporated a broader range of secondary outcomes, including serum urea, urinary creatinine, proteinuria, and albuminuria.

The included RCTs were generally well designed, as reflected by the risk-of-bias assessment. Notably, four trials [21, 28, 38, 43] evaluated creatine supplementation for periods longer than 6 months, addressing important gaps regarding its long-term safety. An additional strength of the included studies is the careful documentation of creatine formulations, ensuring the use of products appropriately tested and verified according to industry standards.

Despite these strengths, several limitations should be acknowledged. Although cystatin C is considered one of the most appropriate biomarkers for kidney function assessment [11, 40, 68], it was evaluated in only two trials: Gualano (2008) and Bender (2008) [21, 40]. In the former, cystatin C levels decreased following creatine supplementation, whereas in the latter no significant changes were observed, even after 2 years of follow-up. Studies investigating creatine supplementation in individuals with non-dialysis CKD (stages 1–5) remain absent from the current evidence base. This represents a critical gap, as this population carries the greatest theoretical risk from potential creatine-induced renal harm, and our safety conclusions cannot be directly extrapolated to this subgroup. Adequately powered trials in non-dialysis CKD are needed before broader clinical recommendations can be made. The available hemodialysis trials, while methodologically sound, were limited by small sample sizes, precluding definitive risk exclusion.

Furthermore, the enforcement of rigorous testing and certification standards is essential to ensure dietary supplement purity. Finally, although this analysis supports the kidney safety of creatine supplementation, creatine use is frequently accompanied by the concomitant consumption of other substances, whether legal or illicit [69]. In such settings, observed kidney effects should be interpreted within the broader context of polypharmacy.

## Conclusion

This study indicates that creatine supplementation does not adversely affect kidney function in adults, including individuals with pre-existing CKD receiving hemodialysis. No clinically meaningful changes indicative of kidney injury were observed across kidney-related parameters, such as serum creatinine, urea, proteinuria, urinary creatinine, or GFR. These findings further support the kidney safety of creatine supplementation, even with long-term use. However, among individuals with advanced CKD, additional well-designed clinical trials remain necessary to confirm these results and inform future clinical recommendations.

## Supplementary Information

Below is the link to the electronic supplementary material.

Supplementary file1 (DOCX 3294 KB)

## Data Availability

No datasets were generated or analysed during the current study.

## Declarations

### Statement of significance

This study represents the most comprehensive meta-analysis on the topic to date (*n* = 26 RCTs) and is the first to rigorously stratify glomerular filtration rate results by measurement method (creatinine-based vs. Cr-EDTA), effectively isolating methodological artifacts from true kidney injury. Unlike previous reviews, we also incorporated a wider range of kidney health markers, such as serum urea and albuminuria, to provide a robust safety assessment for both healthy individuals and patients with chronic kidney disease.
