# Supplementary material for: Impact of creatine supplementation on kidney health: a systematic review and meta-analysis
Source: Int Urol Nephrol. 2026 Jul 27;58(12):5423–34. doi: 10.1007/s11255-026-05287-x (PMC13768353; doi:10.1007/s11255-026-05287-x)
Supplement: Supplementary file 1 [file 11255_2026_5287_MOESM1_ESM.docx]

**Supplemental Online Content**

**eTable 1.** Search Strategy

**eTable 2.** Creatine supplementation protocols across included studies

**eFigure 2.** Serum creatinine - Short Term Subgroup Leave-One-Out Meta-Analysis

**eFigure 3.** Serum creatinine - Short Term Subgroup Baujaut Plot

**eFigure 4.** Serum creatinine - Load + Maintenance Subgroup Leave-One-Out Meta-Analysis

**eFigure 5.** Serum creatinine - Load + Maintenance Subgroup Baujaut Plot

**eFigure 6.** Serum creatinine - Long Term Subgroup Leave-One-Out Meta-Analysis

**eFigure 7.** Serum creatinine - Long Term Subgroup Baujaut Plot

**eFigure 8.** Effect Size by Physical Activity Status on Load + Maintenance Subgroup

**eFigure 9.** BMI vs Effect size on Load + Maintenance Subgroup

**eFigure 10.** Meta-regression: BMI and Physical Activity Status on Load + Maintenance Subgroup

**eFigure 11.** Moderator Effects Comparison Between BMI and Physical Activity Status on Load + Maintenance Subgroup

**eFigure 12.** Partial Effect of BMI on Effect Size on Load + Maintenance Subgroup

**eFigure 13.** Serum Creatinine Trial Sequential Analysis

**eFigure 14.** Urinary Creatinine Forest Plot

**eFigure 15.** Proteinuria Forest Plot

**eFigure 16.** Albuminuria Forest Plot

**eFigure 17.** Urinary creatinine - Leave-One-Out Meta-Analysis

**eFigure 18.** Urinary creatinine - Baujaut Plot

**eFigure 19.** Albuminuria Leave-One-Out Meta-Analysis

**eFigure 20.** Albuminuria Baujaut Plot

**eFigure 21.** Risk of Bias Assessment

**eTable 3.** Summary of findings and certainty of evidence assessment.

**eFigure 22.** Funnel Plot for Serum Urea

**eFigure 23.** Funnel Plot for Serum Creatinine

**eFigure 24.** Funnel Plot for Glomerular Filtration Rate

**eTable 1. Search Strategy**

| MEDLINE | ("creatine supplementation"[TIAB] **OR** "creatine" [TIAB]) **AND** ("renal function" **OR** "kidney function" **OR** "renal insufficiency" **OR** "kidney disease" **OR** "chronic kidney disease" **OR** "CKD" **OR** "kidney impairment") **NOT** ("creatine kinase"[TIAB] **OR** "creatine-phosphokinase"[TIAB] **OR** "CK" [TIAB]) |
| --- | --- |
| Embase | ('creatine supplementation':ti,ab **OR** 'creatine':ti,ab) **AND** ('renal function' **OR** 'kidney function' **OR** 'renal insufficiency' **OR** 'kidney disease' **OR** 'chronic kidney disease' **OR** 'CKD' **OR** 'kidney impairment') **NOT** ('creatine kinase':ti,ab **OR** 'creatine-phosphokinase':ti,ab **OR** 'CK':ti,ab) |
| Cochrane | ("creatine supplementation":ti,ab **OR** "creatine":ti,ab) **AND** ("renal function" **OR** "kidney function" **OR** "renal insufficiency" **OR** "kidney disease" **OR** "chronic kidney disease" **OR** "CKD" **OR** "kidney impairment") **NOT** ("creatine kinase":ti,ab **OR** "creatine-phosphokinase":ti,ab **OR** "CK":ti,ab) |
| Google Scholar | allintitle: creatine supplementation renal **OR** kidney |

**eTable 2. Creatine supplementation protocols across included studies**

| **Study** | **Creatine form** | **Patients** | **Route of administration** | **Protocol** | **Follow-up (days)** |
| --- | --- | --- | --- | --- | --- |
| Almeida, 2013 | monohidrated | males under training program | oral | 0.3 g/kg/d x 7d | 7 |
| Alves, 2013 | monohidrated | women with fibromyalgia | oral | 20 g/d x 5 d + 5 g/d x 25 d | 30 |
| Armentano, 2007 | monohidrated | military | oral | 5 g/d x 6 d + 20 g/d x 8 d | 14 |
| Bender, 2008 | monohidrated | Parkinson’s disease patients | oral | 20 g/d x 6 d + 2 g/d x 180 d + 4 g/d x 540 d | 726 |
| Bennet, 2001 | monohidrated | military | oral | 20 g/d x 6 d + 6 g/d x 28 d | 34 |
| Carvalho 0.03g/kg, 2011 | monohidrated | males under training program | oral | 20 g/d x 7 d + 0.03 g/kg/d x 49 d | 56 |
| Carvalho 5g, 2011 | monohidrated | males under training program | oral | 20 g/d x 7 d + 5 g/d x 49 d | 56 |
| Dalton, 2017 2g CNL | nitrate | healthy adults | oral | 2 g/d x 7 d | 7 |
| Dalton, 2017 4g CNH | nitrate | healthy adults | oral | 4 g/d x 7 d | 7 |
| de Oliveira Vilar Neto, 2020 3g | monohidrated | healthy males | oral | 3 g/d x 35 d | 35 |
| de Oliveira Vilar Neto, 2020 5g | monohidrated | healthy males | oral | 5 g/d x 35 d | 35 |
| Domingues, 2020 | monohidrated | adults with PAD | oral | 20 g/d x 7 d + 5 g/d x 49 d | 56 |
| Galvan, 2016 CM | monohidrated | healthy males | oral | 5 g/d x 28 d | 28 |
| Galvan, 2016 CNH | nitrate | healthy males | oral | 3 g/d x 28 d | 28 |
| Galvan, 2016 CNL | nitrate | healthy males | oral | 1.5 g/d x 28 d | 28 |
| Gotshalk, 2002 | monohidrated | older men | oral | 0.3 g/kg/d x 7 d | 7 |
| Groeneveld, 2005 | monohidrated | ALS patients | oral | 10 g/d x 310 d | 310 |
| Gualano, 2008 | monohidrated | healthy males | oral | 0.3 g/kg/d x 7 d + 0.15 g/kg/d x 77 d | 84 |
| Gualano, 2011 | monohidrated | DM2 adults | oral | 5 g/d x 84 d | 84 |
| Hayashi, 2014 | monohidrated | cSLE patients | oral | 0.1 g/kg/d x 84 d | 84 |
| Kreider, 1998 | monohidrated | male athletes | oral | 15 g/d x 28 d | 28 |
| Lobo, 2015 | monohidrated | older women | oral | 1 g/d x 360 d | 360 |
| **Study** | **Creatine form** | **Patients** | **Route of administration** | **Protocol** | **Follow-up (days)** |
| Lugaresi, 2013 | monohidrated | healthy males | oral | 20 g/d x 5 d + 5 g/d x 84 d | 89 |
| Lyoo, 2012 | monohidrated | women with major depressive disorder | oral | 3 g/d x 7 d + 5 g/d x 49 d | 56 |
| Marini, 2020 | monohidrated | hemodialysis patients | oral | 20 g/d x 7 d + 5 g/d x 21 d | 28 |
| Marini, 2024 | monohidrated | hemodialysis patients | oral | 5 g/d x 360 d | 360 |
| Mihic, 2000 | monohidrated | healthy adults | oral | 20 g/d x 5 d | 5 |
| Neves, 2011 | monohidrated | postmenopausal women | oral | 20 g/d x 7 d + 5 g/d x 77 d | 84 |
| Robinson, 2000 - CrLoad | monohidrated | healthy patients | oral | 20 g/d x 5 d | 5 |
| Robinson, 2000 - CrLoad+G | monohidrated | healthy patients | oral | 20 g/d x 5 d | 5 |
| Robinson, 2000 - CrMaint | monohidrated | healthy patients | oral | 20 g/d x 5 d + 3 g/d x 56 d | 61 |
| Robinson, 2000 - CrMaint+Ex | monohidrated | healthy patients | oral | 20 g/d x 5 d + 3 g/d x 56 d | 61 |
| Solis, 2016 | monohidrated | JDM patients | oral | 0.1 g/kg/d x 84 d | 84 |
| **Study** | **Creatine form** | **Patients** | **Route of administration** | **Protocol** | **Follow-up (days)** |
| Volek, 2001 | monohidrated | healthy males | oral | 0.3g/kg/d x 7 d | 7 |

BMI: body mass index; ALS: amyotrophic lateral sclerosis; DM2: diabetes mellitus type 2; JDM: juvenile dermatomyositis; cSLE: childhood-onset systemic lupus erythematosus; PAD: peripheral artery disease; CM: creatine monohidrated; CNH: creatine nitrate high dose; CNL: creatine nitrate low dose; CrLoad: creatine load; CrLoad + Gl: creatine load + glucose; CrMaint: creatine maintenance; CrMaint + Ex: creatine maintenance + exercise

**eFigure 2. Serum creatinine - Short Term Subgroup Leave-One-Out Meta-Analysis**

**eFigure 3. Serum creatinine - Short Term Subgroup Baujaut Plot**

**eFigure 4. Serum creatinine - Load + Maintenance Subgroup Leave-One-Out Meta-Analysis**

**eFigure 5. Serum creatinine - Load + Maintenance Subgroup Baujaut Plot**

**eFigure 6. Serum creatinine - Long Term Subgroup Leave-One-Out Meta-Analysis**

**eFigure 7. Serum creatinine - Long Term Subgroup Baujaut Plot**

**eFigure 8. Effect Size by Physical Activity Status on Load + Maintenance Subgroup**

**eFigure 9. BMI vs Effect size on Load + Maintenance Subgroup**

**eFigure 10. Meta-regression: BMI and Physical Activity Status on Load + Maintenance Subgroup**

**eFigure 11. Moderator Effects Comparison Between BMI and Physical Activity Status on Load + Maintenance Subgroup**

**eFigure 12. Partial Effect of BMI on Effect Size on Load + Maintenance Subgroup**

**eFigure 13. Serum Creatinine Trial Sequential Analysis**

**eFigure 14. Urinary Creatinine Forest Plot**

**eFigure 15. Proteinuria Forest Plot**

**eFigure 16. Albuminuria Forest Plot**

**eFigure 17. Urinary creatinine - Leave-One-Out Meta-Analysis**

**eFigure 18. Urinary creatinine - Baujaut Plot**

**eFigure 19. Albuminuria Leave-One-Out Meta-Analysis**

**eFigure 20. Albuminuria Baujaut Plot**

**eFigure 21. Risk of Bias Assessment**

**eTable 2. Summary of findings and certainty of evidence assessment.**

| **Outcome** | **No participants** | | **Mean difference [CI 95%]** | ***P*-value** | **Certainty of Evidence (GRADE)** |
| --- | --- | --- | --- | --- | --- |
|  | **Creatine** | **Placebo** |  |  |  |
| Serum creatinine - long-term supplementation subgroup | 180 | 150 | 0.0880 [0.0305; 0.1456] | 0.003 | ⨁⨁⨁⨁ High^b^ |
| Serum creatinine - patients undergoing hemodialysis | 33 | 35 | 2.7355 [2.2439; 3.2271] | <0.001 | ⨁⨁⨁⨁ High |
| Serum urea | 401 | 309 | -0.5238 [-2.9771; 1.9295] | 0.676 | ⨁⨁⨁⨁ High |
| GFR | 132 | 126 | -2.19 [-9.18; 4.80] | 0.539 | ⨁⨁⨁◯ Moderate^c,d^ |
| Albuminuria | 98 | 86 | 0.4084 [-2.0869; 2.9037] | 0.748 | ⨁◯◯◯ Very low^e,f^ |
| Proteinuria | 71 | 73 | 0.0090 [-0.0279; 0.0459] | 0.633 | ⨁⨁⨁◯ Moderate^a^ |
| Urinary creatinine | 59 | 59 | -0.0581 [-0.1957; 0.0795] | 0.408 | ⨁⨁⨁◯ Moderate^a^ |

CI: confidence interval; MD: mean difference

#### Explanations

a. The confidence interval crosses the line of no effect, including both potential benefit and harm, indicating serious imprecision.

b. Although initial heterogeneity was moderate (I² = 47.3%), sensitivity analysis using the leave-one-out method resolved the inconsistency, supporting a consistent direction of effect across studies.

c. Although initial heterogeneity was moderate (I² = 35.2%), sensitivity analysis using the leave-one-out method resolved the inconsistency, supporting a consistent direction of effect across studies.

d. The confidence interval crosses the line of no effect, including both potential benefit and harm, indicating serious imprecision.

e. Substantial and unexplained heterogeneity was observed across studies (I² = 76%), with considerable variation in the direction and magnitude of effects, leading to very serious inconsistency.

f. The confidence interval crosses the line of no effect, including both potential benefit and harm, indicating serious imprecision.

**eFigure 22.** Funnel Plot for Serum Urea

**eFigure 23.** Funnel Plot for Serum Creatinine

**eFigure 24.** Funnel Plot for Glomerular Filtration Rate
